# Second Skin Enabled by Advanced Electronics

**DOI:** 10.1002/advs.201900186

**Published:** 2019-04-01

**Authors:** Jin Young Oh, Zhenan Bao

**Affiliations:** ^1^ Department of Chemical Engineering Kyung Hee University Yongin 17104 Republic of Korea; ^2^ Department of Chemical Engineering Stanford University Stanford CA 94305 USA

**Keywords:** bioelectronics, functional devices, materials science, stretchable electronics, wearable electronics

## Abstract

Electronic second skin is touted as the next interface to expand applications of electronics for natural and seamless interactions with humans to enable smart health care, the Internet of Things, and even to amplify human sensory abilities. Thus, electronic materials are now being actively investigated to construct “second skin.” Accordingly, electronic devices are desirable to have skin‐like properties such as stretchability, self‐healing ability, biocompatibility, and biodegradability. This work reviews recent major progress in the development of both electronic materials and devices toward the second skin. It is concluded with comments on future research directions of the field.

## 1 Introduction

Rapid development in science and technology has drastically reduced the spatial separation between human and electronic devices from meter scales for “stationary” devices (e.g., personal computers) to centimeter scales for portable devices (e.g., cell phones) and, finally, to millimeter scales for wearable devices (e.g., smart watches).^1^ In the near future, this separation distance may be even eliminated, as the devices can now be mounted directly on the skin or even implanted inside the human body.^2^ As such, these wearable devices will require new capabilities such as stretchability, self‐healing, biocompatibility, and biodegradability.^3^

This new electronic device platform has been termed as the “second skin.” In essence, the second skin is fully integrated electronic system composed of multifunctional electronic components operating on or inside the body to provide smart health care, enhanced capabilities and prevention or treatment of disease.^4^ The second skin could also serve as a module to connect our human body to the internet, thereby allowing integration of humans to the “Internet of Things” for next‐generation wireless communication.^5^ Thus, the second skin may be viewed as an artificial body part that can be used to improve our everyday quality‐of‐life.

This review covers recent advances in electronic materials and devices for use in the second skin. Briefly, in Section 2, we highlight several important skin‐like capabilities incorporated into electronic material, such as stretchability, self‐healing, biocompatibility, and biodegradability. In Section 3, we describe several notable functional devices, such as sensors, integrated circuits, displays, and power supplies, for the second skin. In Section 4, we conclude with our perspectives of the second skin future directions.

**Figure 1:**
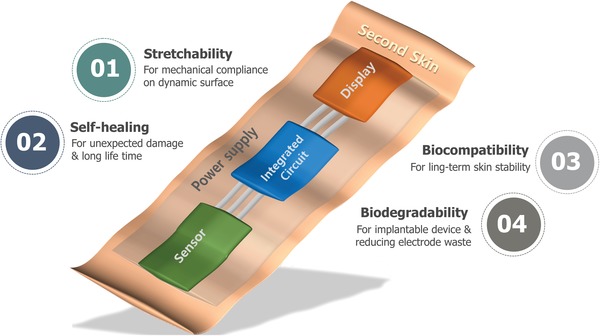
Schematic illustration of structure of second skin composed of functional devices: sensor, integrated circuit, display, and power supply. Required novel capabilities of electronic materials for the second skin include stretchability, self‐healing capability, biocompatibility, and biodegradability.

The general structure of a second skin is shown in **Figure** **1**, and it is composed of various functional devices, including: multifunctional sensors to obtain various biosignals from the human body, integrated circuits to electrically process the signals, displays to convey the analyzed data and feedback to the user, and a power supply.

For operation on or inside a human body, the electronic materials of the second skin need to process four novel capabilities: (i) stretchability to allow the device to accommodate strains generated by dynamic deformation due to human movement without delaminating or failing mechanically, (ii) self‐healing ability to prevent extend its life time should mechanical failure occurs, (iii) biocompatibility for long‐term stability, and (iv) biodegradability, so that an implanted device can disintegrate without surgery, and to reduce electronic waste. Recent advances have imparted several of these desired properties to electronic materials and devices and will be elaborated below.

## 2 Advanced Materials for Second Skin

New classes of electronic materials for next‐generation electronics have now been shown to possess important parameters in the stretchability, self‐healing capability, biocompatibility, and biodegradability, thus mimicking the characteristics of biomaterials.

### 2.1 Stretchability

Stretchability of electronic materials is essential for conformal integration of electronic components on skin, as it will improve the overall robustness to avoid mechanical failure incurred during various human motions. Various concepts have been reported to develop electronic materials that have either extrinsic^6^ or intrinsic^7^ stretchabilities. There are three main strategies: strain engineering, use of hybrid composites, and dynamic intermolecular interaction.

Strain‐engineering has typically been used to impart extrinsic stretchability to conventional electronic materials that are usually rigid and brittle.^8^ Various geometric structures are designed to release applied strain. The main advantage of this approach is that it enables the use of well‐developed conventional classic electronic materials, such as silicon, metals, and oxides, where existing knowledge in materials and device fabrications can be leveraged.^9^ Buckling and wrinkling are simple and effective methods to form wavy structures using prestrained elastomer by first releasing the prestrain.^10^ For example, a wavy single‐crystal thin silicon ribbon formed on a prestrained elastomer can be reversibly stretched and released without being mechanically damaged.[qv: 8c] A stretchable transistor and p–n diode made of wavy silicon ribbon have been demonstrated on elastomer. This approach has even been applied to make crumble organic circuit and organic/inorganic solar cells.[qv: 8e,45]

**Figure 2:**
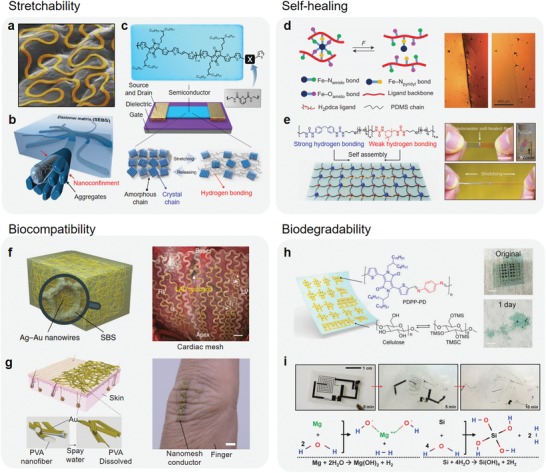
Advanced electronic materials. Stretchability: a) strain engineering of serpentine gold fractal design on human skin replica. Reproduced with permission.^11^ Copyright 2013, Nature Publishing Group. b) Hybrid composite using nanoconfinement effect of semiconducting polymer nanowire in elastomer. Reproduced with permission.[qv: 12a] Copyright 2017, AAAS. c) Molecular design of conjugated polymer by using dynamic bonding. Reproduced with permission.[qv: 13a] Copyright 2016, Nature Publishing Group. Self‐healing: d) autonomous self‐healing elastomer using metal coordination. Reproduced with permission.[qv: 18a] Copyright 2016, Nature Publishing Group. e) Tough and water‐insensitive self‐healing elastomer. Reproduced with permission.[qv: 18b] Copyright 2018, Wiley‐VCH. Biocompatibility: f) biocompatible nanowire composite and implanted cardiac mesh made of the composite. Reproduced with permission.^22^ Copyright 2018, Nature Publishing Group. g) On‐skin nanomesh electronics. Reproduced with permission.^35^ Copyright 2016, Nature Publishing Group. Biodegradability: h) degradable semiconducting polymer. Reproduced with permission.^29^ Copyright 2017, National Academy of Sciences. i) Bioresorbable electronic device composed of silicone and magnesium. Reproduced with permission.[qv: 30a] Copyright 2012, AAAS.

Serpentine structure inspired by fractal design is a widely used strain‐engineering structure for interconnector.^8^ For example, a serpentine gold interconnect (**Figure** **2**a) showed conformal contact on human skin.^11^ The design of the serpentine structure allows biaxial strain. It was also useful to connect rigid island electronic components on an elastomer, and thereby enabling high‐performance stretchable electronics using classic electronic components.

Many hybrid composites of elastomer and nano‐ and microelectronic materials, based on percolation theory of electronic materials in elastomers, have been recently reported.^12^ Various nanomaterials, such as nanoparticles, nanoflakes, and nanowires, have been widely utilized as the filler materials for stretchable conductors. However, formation of semiconducting percolation paths in elastomer remains challenging. Much fewer advances have been made in the development of stretchable semiconductors than in stretchable conductors.[qv: 12c]

Nanoconfinement effect can alter the mechanical properties of polymer materials and recent exploitation of this effect represented a breakthrough for stretchable polymer semiconductors. Specifically, the nanoconfinement effect was investigated to form a stretchable semiconductor by spontaneous phase separation to form 1D semiconducting polymer nanostructures in an elastomer matrix (Figure 2b).[qv: 12a] The 1D nanoconfined semiconducting polymer structures were observed to possess enhanced polymer chain dynamics and decreased crystallinity. The glass transition temperature of the 1D nanostructure was much lowered than the neat polymer thin film. To form a percolating polymer semiconductor network with high carrier mobility (*µ*) similar to a pure film, choosing polymer semiconductor and elastomer with match surface energy was important Moreover, the elastic matrix was able to impart elasticity and thus preventing crack propagation through soft interface. The semiconducting film with nanoconfined polymer network can be stretched up to 100% strain while maintaining its *µ* to be >1 cm^2^ V^−1^ s^−1^, a value comparable of amorphous silicon.

Molecular design to provide intrinsic stretchability to polymer electronic materials without compromising electronic performance is challenging, but highly attractive for truly stretchable electronics.^13^ Intrinsically stretchable polymers can be obtained by incorporating functional molecular blocks to provide dynamic molecular interaction. Dynamic bonding between polymer chains can impart stretchability to insulating polymers and semiconducting polymers. Noncovalent dynamic crosslinking enables energy dissipation by breaking the dynamic bonding during strain, while the bonds can subsequently reform when the strain is released.[qv: 13a] For example, noncovalent dynamic crosslinking can be achieved through weak hydrogen bonding by attaching (2,6‐pyridine dicarboxamide, PDCA) moieties to a 3,6‐di(thiophen‐2‐yl)‐2,5‐dihydropyrrolo[3,4‐c]pyrrole‐1,4‐dione conjugated polymer backbone (Figure 2c).[qv: 13a] Incorporation of non‐conjugated PDCA moieties at ≈10 mol% effectively reduced the elastic modulus of the polymer by almost ten times compared to the fully conjugated one. Furthermore, the resulting material became stretchable to above 100% strain. Stretching and alignment of polymer chains in the amorphous regions and partial breakages of the crystalline domains were also formed to have contributed to the improved stretchability, together with energy dissipation through dynamic bonding.[qv: 13a]

### 2.2 Self‐Healing

Skin‐mountable functional electronic devices akin to another skin on a human body may ultimately have self‐healing capability.[qv: 3c] Researchers have already obtained self‐healing electronic materials through various strategies of material design.^14^

There are two basic types of self‐healing materials: extrinsic or intrinsic self‐healing systems. Extrinsic self‐healing materials generally incorporate capsules or vascular tubes filled with reactive chemicals, which are then released by fractures at the damaged regions.^15^ The released agents trigger polymerization or crosslinking to enable self‐healing of the fractures. However, such systems typically can be healed only once in the same location. On the other hand, intrinsically self‐healing systems are desirable due to their simplicity and repeatability.^16^ Dynamic intermolecular interactions, i.e., hydrogen bonding, metal coordination, π–π interaction, and electrostatic interaction, are widely used as the reversible bondings for intrinsic self‐healing.^17^ Multiple dynamic bonds consisting of more than two different dynamic bonding strengths in a material have been observed to achieve efficient autonomous self‐healing at even low temperature (<0 °C) (Figure 2d).^18^ A highly stretchable and autonomous self‐healable elastomer with different dynamic bonding strengths was demonstrated by incorporating a metal coordination moiety into an elastic polymer backbone.[qv: 18a] Poly(dimethylsiloxane) (PDMS) polymer chains was dynamically crosslinked by a Fe(III)‐2,6‐PDCA coordination complex. The metal coordination complex has three dynamic bonding strengths (strong, middle, and weak), so it can respond in several ways to breakage and reformation during stretching and self‐healing. The self‐healing elastomer is stretchable over 10 000%, autonomously self‐healable below room temperature, and has high dielectric strength. This multiple dynamic bonding system may also helped to overcome the drawbacks of self‐healing elastomers (e.g., low mechanical property, high moisture sensitivity). A dual‐hydrogen‐bonding system has achieved highly a tough and water‐insensitive self‐healing elastomer (Figure 2e).[qv: 18b] Strong and weak dynamic‐bonding moieties were incorporated into PDMS polymer chain. The strong hydrogen bonding provides high elasticity for good mechanical property, and the weak hydrogen bonding allows energy dissipation by the elastomer for high stretchability. This self‐healing elastomer was highly tough (fracture energy measured at 12 kJ m^−2^) and autonomously self‐healable even in artificial sweat.

### 2.3 Biocompatibility

Electronic devices mounted on human skin or organs should be biocompatible to avoid immune reactions.^19^ Various biocompatible materials have been developed for biomedical applications, but most of these materials are insulators.^20^ On the other hand, electronic components require conductors and semiconductors.^21^ Recently, a highly conductive, stretchable, and biocompatible composite was reported for bioelectronic applications (Figure 2f). It is composed of an Ag–Au core‐sheath nanowire and poly(styrene–butadiene–styrene) elastomer.^22^ A thick Au layer deposited on ultralong Ag nanowire protects the Ag from oxidation and impedes leaching of Ag ions, so the composite can be both biocompatible and conductive. The composite has high conductivity (41850 S cm^−2^) and optimized stretchability (266%). Devices fabricated using the composites successfully recorded electrophysiological signals on human skin and swine heart.

Breathable electronic devices may also improve biocompatibility of electronic devices on skin.^23^ On‐skin electronics with nanomesh allow for hypoallergenic electronic devices (Figure 2g).^24^ To form an Au nanomesh on skin, Au was first thermally evaporated onto electro spun poly(vinyl alcohol) (PVA) nanofibers. Next, the PVA nanomesh frame was dissolved by spraying water on the skin. The Au nanomesh was observed to be adhered to skin and stretchable up to 10% strain, while maintaining its electrical property. The nanomesh‐based device efficiently reduced the contact area between the device and the skin. It also allowed the skin better breathability through the mesh. As a result, skin irritation and user discomfort were then minimized. Direct adhesion of the Au nanomesh to the skin provided an excellent interface for measuring skin temperature, pressure, touch, and even electromyograms.

### 2.4 Biodegradability

Biodegradable electronic materials capable of breaking down into harmless components can drastically reduce pollution from electronic waste.^25^ Biodegradability would also be useful in implantable biomedical devices to enable nonsurgical removal after they have served their purposes.^26^

Biodegradable electronic materials can be broadly categorized into organic materials (especially polymers) and inorganic materials.^27^ For example, cellulose, silk fibroin, and shellac are biodegradable polymers that are present in nature. Synthetic biodegradable polymers (e.g., PVA, polylactic acid, polycaprolactone, polyurethane, polyethylene glycol, poly lactic‐*co*‐glycolic acid) have been utilized for biomedical applications.^28^ However, all of these materials are electrically insulating. Thus, development of biodegradable electronic devices has limited to using only a few selected biodegradable substrates and physiologically compatible metals. Other electronic materials, especially semiconductors, must be also developed. Recently, a degradable semiconducting polymer was demonstrated using reversible imine chemistry (Figure 2h) and the polymer was observed to disintegrate completely in 30 days.^29^ Using a cellulose substrate, the researchers fabricated an ultralightweight (≈2 g m^−2^) and ultrathin (<1 µm) pseudocomplementary metal‐oxide‐semiconductor composed of polymer transistors for transient electronics. Again, this device was observed to be also fully disintegrable and biocompatible.

Inorganic electronic materials were also recently developed using corrosion chemistry.^30^ Some inorganic electronic materials (e.g., Si, SiO_2_, Mg, MgO) can be dissolved through hydrolysis in water (Figure 2i).[qv: 30a] The reaction kinetics shown to be strongly dependent on pH, and the overall transient time was controlled by the size of a silicon nanomembrane and encapsulation layer. Silicon electronics for biomedical applications have been thus fabricated based on these characteristics. Specifically, a transient electronic platform including transistors, diodes, inductors, capacitors, and resistors, was designed to dissolve in body over time. It was then used in a bioresorbable implant for thermal therapy and as a brain sensor. Despite the high promises of biodegradable electronics, excessive dosage of inorganic materials introduced into human body may be toxic and must hence be restricted. Furthermore, their mechanical rigidity at implant/tissue interface also need to be improved.

## 3 Advanced Devices for Second Skin

The second skin will be composed of functional electronic devices to interact with humans on their skin or within the human body.[qv: 2a] Functional devices can be classified as sensors, integrated circuits, displays, and power supplies for system‐level applications. We introduce here key functional devices for the second skin.

### 3.1 Sensor

Our skin consists of epidermis, dermis, and hypodermis, and with embedded sweat glands, nerve endings, blood vessels, and muscle. The outside epidermal layer is an ideal surface for health monitoring and theragnosis.^31^ Thus, skin‐mounted sensors are being actively developed for biomedical applications.^32^

**Figure 3:**
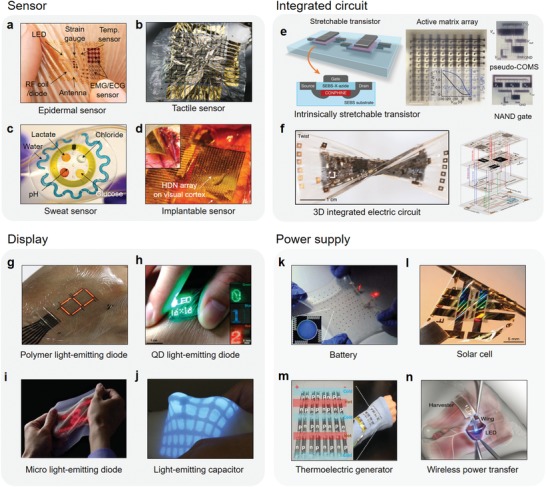
Advanced electronic devices. Sensor: a) epidermal sensor. Reproduced with permission.^33^ Copyright 2011, AAAS. b) Tactile sensor. Reproduced with permission.[qv: 34a] Copyright 2013, Nature Publishing Group. c) Sweat sensor. Reproduced with permission.^37^ Copyright 2015, AAAS. d) Implantable sensor. Reproduced with permission.^40^ Copyright 2011, Nature Publishing Group. Integrated circuit: e) intrinsically stretchable electronic circuit. Reproduced with permission.^42^ Copyright 2018, Nature Publishing Group. f) 3D integrated circuit. Reproduced with permission.^43^ Copyright 2018, Nature Publishing Group. Display: g) polymer light‐emitting diode. Reproduced with permission.^45^ Copyright 2016, AAAS. f) Quantum dot light‐emitting diode. Reproduced with permission.[qv: 46a] Copyright 2017, Wiley‐VCH. i) Microlight‐emitting diode. Reproduced with permission.[qv: 44d] Copyright 2018, Someya group. j) Light‐emitting capacitor. Reproduced with permission.^47^ Copyright 2016, AAAS. Power supply: k) battery. Reproduced with permission.^50^ Copyright 2013, Nature Publishing Group. i) Solar cell. Reproduced with permission.^54^ Copyright 2018, Nature Publishing Group. m) Thermoelectric generator. Reproduced with permission.^57^ Copyright 2016, The Royal Society of Chemistry. n) Wireless power transfer. Reproduced with permission.^59^ Copyright 2015, Nature Publishing Group.

A system‐level epidermal sensor to measure biopotentials, temperature, and even strain has been recently reported (**Figure** **3**a).^33^ An epidermal patch that was strain‐engineered into a serpentine structure was observed to be stretchable and adhesive to skin. This report demonstrated the potential of skin to measure various biosignals and can be thus utilized as an unusual type of electronic controller to the obtain electromyogram information. The epidermal patch was useful in diagnosis of movement disorders, and in therapy as a multifunctional drug‐delivery system.

Ultralightweight design of a skin‐mountable sensor can make it imperceptible to users.^34^ Imperceptible tactile sensor has been developed using plastic electronics for electronic skin, prosthetics, and robotics (Figure 3b).[qv: 34a] To improve the quality of obtained signals is to minimize electrical cross‐talk. Thus, the tactile sensor was designed to interconnect with an active matrix array on an organic transistor. The tactile sensor was fabricated on ultrathin film (1 µm), was lightweight (3 g m^−2^) and ultraflexible, so it can be attached to skin or any dynamic surfaces. This system was then used with composite nanofibers of carbon nanotube and graphene in a transparent bending‐insensitive pressure sensor for medical applications.^35^

Secreted human sweat can convey useful bioinformation. Hence, diagnostics based on sweat can be an effective noninvasive monitoring method to provide insights into the health of a human.^36^ Despite its advantages, sweat diagnostics has been limited to laboratory or hospital settings.^36^ A skin‐mountable sweat sensor was demonstrated to expand the usability and convenience of sweat‐based diagnostics (Figure 3c).^37^ The sweat sensor was fabricated with a stretchable microfluidic system and colorimetric sensor to capture, store, and analyze sweat. The sensor can measure quantitative values of sweat rate, total sweat loss, pH, and concentrations of chloride and lactate, and can send the information wirelessly using near field communication technology. Another type of skin‐mountable sensor composed of a flexible integrated sensing array was also developed for in situ sweat analysis.^38^ The fully integrated system of the sensor can simultaneously and selectively measure sweat metabolites and electrotypes, and even body temperature while wirelessly sending the collected information directly to the users.

Implantable sensors to derive high‐quality biosignals from internal organs such as brain, heart, and blood vessels, will be more beneficial than skin‐mounted sensors.^39^ A flexible multiplexed electrode array for electrocorticogram measurement (Figure 3d) uses a high‐density multiplexed sensor array to monitor microscale brain activities over large areas in vivo and theragnosis of disorders.^40^ Electronic stents in blood vessel have also been demonstrated as smart implants for in vivo health monitoring and treatment of endovascular diseases.^41^ Specifically, a bioresorbable electronic stent integrated with sensor, memory, wireless communication module, and drug delivery systems has been shown to demonstrate multifunctional capabilities in sensing flows, monitor temperature, store data, along with the ability to transmit wireless power or data.^41^ Furthermore, it also showed efficient hyperthermia therapy by using bioinert therapeutic nanoparticles in the endovascular system.

### 3.2 Integrated Circuits

Intrinsically stretchable electronic circuits are highly desirable for applications in skin‐mounted devices. An intrinsically stretchable active matrix array was recently achieved by developing a scalable fabrication of intrinsically stretchable transistors (Figure 3e).^42^ This is important for high‐density device arrays for integrated circuits of functional electronics. The fabrication process involves developing photopatterning processes for dielectric, semiconductor. A high‐density transistor (347 transistors cm^−2^) array was realized. The stretchable transistor array was fabricated into analogue and digital circuits, and an active matrix for a tactile sensor array.

3D integrated stretchable electronics is a new direction to achieve high‐density devices which would have required a significant larger area with single‐layer formats. A framework for 3D integrated stretchable electronic circuit systems was demonstrated by transfer printing of predesigned stretchable circuits.^43^ The 3D integrated stretchable electronic system (Figure 3f) consisted of four electronic circuits. Each layer had individual functions, and each layer was connected using the vertical interconnect access process. The 3D integrated stretchable system was then used in stretchable, compact multichannel sensors to control a wireless robotic arm for human–machine interfaces.

### 3.3 Displays

Displays enable direct visualization of the processed information to a user. Several types of light‐emitting devices have been demonstrated for skin‐mountable displays.^44^ Organic light‐emitting diodes (OLEDs) have already been developed as a platforms for high‐resolution flexible displays.^45^ An ultraflexible OLED display was recently realized by fabricating the device on ultraflexible polymer substrate (2 µm) (Figure 3g).[qv: 45b] Its ultraflexibility enabled the device to easily adhere to skin with minimal mechanical constrain. In addition, quantum dot light‐emitting diodes (QLEDs) are also strong candidates for wearable displays, due to high color purity, electroluminescence brightness, low operating voltage, and stability in air and under illumination.^46^ An ultrathin (5 µm) QLED for skin‐attachable display (Figure 3h) was integrated with a smart wristwatch enables visualization of various vital information, such as body temperature and motion changes.[qv: 46a] Recently, an elastic display made of nanomesh conductor and micro LED array has been shown to display relevant collected biosignals (Figure 3i).[qv: 44d]

Stretchable light‐emitting capacitors (LECs) are also promising candidates for skin‐mountable displays.^47^ The LEC simple structure and operating mechanism enable easy fabrication. An LEC consists of a composite emitting layer composed of an elastomer and an emitting phosphor powder. The elastic emitting layer is both highly stretchable and deformable under strain, and can display sensory feedback about various stimuli (Figure 3j).^47^ A self‐healing elastomer was used to realize a stretchable self‐healable LEC, which was integrated with electronic skin, and can then be used to generate visualization of collected biosignals for health monitoring.^48^

### 3.4 Power Supply

Development of a robust and stable power supply system for skin‐mountable devices is essential for allowing the devices to function reliably and continuously. Rechargeable batteries are the most obvious choice because they were first commercialized as the power supply for portable electronic devices.^49^ Stretchable batteries were recently achieved through fabrication on elastomer and connected by serpentine interconnects (Figure 3k).^50^ These connected batteries can be stretched up to 300% and be integrated to wireless charging systems. However, rechargeable batteries have a drawback as it may at times overheat, leading to minor explosion. When incorporated in skin‐mountable devices, the batteries are in intimate contact with human body, so any potential the explosion hazards must be prevented before they can be considered as power supplies.^51^ Furthermore, their long recharging time must be minimized to reduce downtime and inconvenience.

Solar cells are promising candidates as wireless power supplies to generate renewable energy for power skin‐mountable devices.^52^ A stretchable GaAs solar cell has been demonstrated on prestrained elastomer. This achievement illustrates the possibility in developing self‐powered electronics.^53^ Recently, self‐powered ultraflexible electronics have been demonstrated using a nanograting‐patterned organic solar cell (Figure 3l).^54^ The fabricated ultrathin organic solar cell is lightweight and ultraflexible, and is conformable to human skin or other tissue. Self‐powered ultraflexible electronic system integrated with the organic solar cell and organic electrochemical transistors was shown to reliably measure cardiac and brain signals on and in the body.^54^

Next, heat energy stemming from human skin can also be utilized as a power source for skin‐mountable devices.^55^ A thermoelectric generator that directly converts heat flux to electricity is attractive as a self‐powered supply that uses generated body heat. A flexible thermoelectric generator based on glass fabric was recently fabricated for wearable self‐powered electronics.^56^ Specifically, Bi_2_Te_3_ (n‐type) and Sb_2_Te_3_ (p‐type) pastes were screen‐printed on glass fabric, and the obtained wearable device was flexible and possessed high output power density. Next, stretchable thermoelectric generators based on transition metal dichalcogenide (TMD) nanosheets (WS_2_: p‐type and NbSe_2_: n‐type) have also been demonstrated (Figure 3m).^57^ A multistacked TMD nanosheet film was shown to maintain its electrical percolation pathway even when stretched and partially torn and fold. The changed morphology of the film was rapidly recovered by a plug‐in contact between the torn regions after the strain was released. The researchers proceeded to demonstrate a prototype self‐powered wristband based on the above TMD nanosheet film.

Wireless power transfer is a useful method to supply energy or power to electronic devices without using a power‐supply module.^58^ In an optogenetic device (Figure 3n), power was transferred wirelessly by using inductive coupling to control neuronal activity of light‐sensitive proteins in a mouse.^59^ This approach was able to address the significant constraints encountered when light was transferred through a long optical fiber when connected to a light source.

## 4 Conclusion and Perspectives

In this review, we highlighted recent developed advanced electronic materials as second skin for next generation wearable electronics. These materials include organic/inorganic materials, polymers, and hybrid composite materials that possesses novel capabilities of stretchability, self‐healing ability, biocompatibility, and biodegradability. These developments allow subsequent fabrication of functional electronic devices to integrate with the human body. The second skin created by such advanced electronics could enable extension of applications of electronics to areas such as health care, sport, and military fields through communicating with our human body as artificial human parts. The second skin could enable humans to become a part of the Internet of Things, a fundamental component of The Fourth Industrial Revolution. Before second skin can be commercialized, several impediments need to be overcome, which includes: (i) the chemical stability of the materials should be improved, (ii) the electronic characteristics of the device must be improved, (iii) electronics must be standardized, and (iv) developing methods to integrate the technologies of all components. We believe that when these problems are addressed, second skin can thus become a critical player in next‐generation electronics.

## Conflict of Interest

The authors declare no conflict of interest.
